# The interplay between cardiac dyads and mitochondria regulated the calcium handling in cardiomyocytes

**DOI:** 10.3389/fphys.2022.1013817

**Published:** 2022-12-02

**Authors:** Lei Liu, Kaiyu Zhou, Xiaoliang Liu, Yimin Hua, Hua Wang, Yifei Li

**Affiliations:** Key Laboratory of Birth Defects and Related Diseases of Women and Children of MOE, Department of Pediatrics, West China Second University Hospital, Sichuan University, Chengdu, China

**Keywords:** calcium handling, mitochondria, cardiomyocytes, heart failure, molecular mechanisms

## Abstract

Calcium mishandling and mitochondrial dysfunction have been increasingly recognized as significant factors involved in the progression procedure of cardiomyopathy. Ca2+ mishandling could cause calcium-triggered arrhythmias, which could enhance force development and ATP consumption. Mitochondrial disorganization and dysfunction in cardiomyopathy could disturb the balance of energy catabolic and anabolic procedure. Close spatial localization and arrangement of structural among T-tubule, sarcoplasmic reticulum, mitochondria are important for Ca2+ handling. So that, we illustrate the regulating network between calcium handling and mitochondrial homeostasis, as well as its intracellular mechanisms in this review, which would be worthy to develop novel therapeutic strategy and restore the function of injured cardiomyocytes.

## Introduction

Cardiomyopathy leads to a substantially elevated risk for morbidity and mortality (Lipshultz et al., 2019). A particular pathogenesis and particular genetic variants, clinical manifestations, alternations in cardiac structures, and changes in heart rhythm are associated with prognosis and are independent risk factors affecting cardiac geometry and function. Among all the patients with cardiomyopathy, children cases are always associated more risk of adverse outcomes. The clinical phenotype of cardiomyopathy is determined by the risk factors involved. Genetic variants in loci encoding sarcomeres, the cytoskeleton, mitochondria, gap junctions, and ion channel proteins predominantly contribute to cardiomyopathy, including Barth syndrome with *Taffazzin* gene mutation (Liu et al., 2021), arrhythmogenic cardiomyopathy (ACM) especially with *DSP*, *DSG2*, *DSC2* and *PKP2* mutations (Austin et al., 2019) and dilated cardiomyopathy (DCM) with *LMNA* mutation (Yang et al., 2022). Although the primary morphofunctions of cardiomyopathy vary by classification, they manifest with systolic dysfunction, clinical heart failure, and arrhythmia at the end stage of disease (Ranjbarvaziri et al., 2021). However, there is still a gap in determining children related cardiomyopathy. Most of researches on cardiomyopathy focused adult disorders in cardiomyocytes. Recently, the studies on cardiomyocytes maturation brought new insights into the mechanisms of cardiomyopathy. Moreover, the application of induced pluripotent stem cell (IPSC) also provided optimal models to explore early onset cardiomyopathies.

The contractility of cardiomyocytes is associated with Ca2+ homeostasis and adenosine tri-phosphate (ATP) provided by mitochondria (Kaasik et al., 2004). Close arrangement and localization of structures such as transverse tubules (T-tubules), the sarcoplasmic reticulum (SR), and mitochondria is favorable for direct functional interactions between these compartments as dyads. A low concentration of Ca2+ from T-tubules induces a large release of Ca2+ from the proximal SR, followed by a transient increase in mitochondrial Ca2+ (Sharma VR et al., 2000). Ca2+ mishandling and mitochondrial dysfunction are significant factors involved in the progression of cardiomyopathy (Dey et al., 2018). Increased myofilament Ca2+ sensitivity has been identified in animal models of and patients with cardiomyopathy. This can lead to changes in intracellular Ca2+ homeostasis and cause calcium-triggered arrhythmias, which can enhance force development and ATP consumption. Mitochondrial disorganization and dysfunction in cardiomyopathy can disturb the balance of energy catabolism and anabolism. Emerging evidence demonstrates that crosstalk between mitochondrial function and calcium handling mediates the pathophysiology of cardiomyopathies and heart failure, which is strictly related to clinical outcomes and survival. Thus, it is critical to understand the regulatory network underlying calcium and mitochondrial homeostasis as well as its intracellular mechanisms to develop novel therapeutic strategies and restore the function of damaged cardiomyocytes.

### T-tubule system remodeling in cardiomyopathy or heart failure

The microarchitecture of cardiac dyads in cardiomyocytes, which was considered as the structural basis of calcium transits in cardiomyocytes. The release of calcium from the SR and efficient ATP production by mitochondria are essential for normal excitation-contraction (EC) coupling. The increase in cytosolic calcium is augmented by calcium release at numerous dyads. T-tubule depolarization is mediated by coupling L-type Ca2+ channels (LTCCs) next to ryanodine receptors (RyRs), which activates RyR2 channels by a small amount of Ca2+ entry through LTCCs and leads to the release of a large amount of calcium mediated by RyR2 (Marks, 2013; Eisner et al., 2017; Seidel et al., 2019). Dyads of atrial cardiomyocytes (AMs) are characterized by high-density axial tubules (ATs), whereas ventricular cardiomyocytes (VMs) in mouse and human have high-density T-tubules with diameters of 100–300 nm (Brandenburg et al., 2016).

Left cardiac hypertrophy induced by transverse aortic constriction leads to the proliferation of AMs and ATs and increases the phosphorylation of RyR2, thereby maintaining a higher signal of cytosolic Ca2+ activity (Brandenburg et al., 2016; Novotova et al., 2020). A remodeling of T-tubules in VMs, including a change in the number of T-tubule components, oblique directions, the diameter and length of T-tubules, and the opening time of T-tubules, has been observed in several animal models of cardiomyopathies and human samples with heart disease (Crocini et al., 2017). Hatano et al. (2015) confirmed that the T-tubule structure is important for the synchrony of Ca2+ release and suggested that mitochondria in the sub-sarcolemmal region might serve to cancel Ca2+ inflow through surface sarcolemma, thereby maintaining equilibrium in the intracellular Ca2+ environment. Thus, the remodeling of T-tubules alters the distance to the junctional SR or sarcolemma, which delays the transduction of calcium. Dyad remodeling is associated with electrical and contractile defects in cardiomyocytes. Ultimately, T-tubule remodeling would be an ideal mechanism for treating heart disease.

**FIGURE 1:**
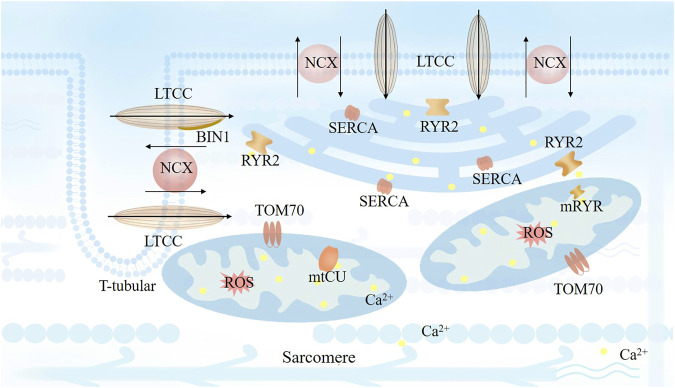
Cardiomyocyte excitation-contraction (EC) coupling based on the close spatial and arrangement localization of the dyads. Transverse tubule (T-tubule) depolarization is mediated by coupling L-type Ca2+ channels (LTCCs) next to ryanodine receptors (RyRs), which activates RyR2 channels by a small amount of Ca2+ entry through LTCCs and lead to a following large amount calcium release mediated by RyR2. Bridging integrator 1 (BIN1) facilitates LTCC localization to T-tubule membrane. The released Ca2+ from SR would be reuptake by SR calcium ATPase (SERCA) on every cardiac cycle to sustain a steady state with stable Ca2+ level.

Bridging integrator 1 (BIN1; also known as amphiphysin 2) facilitates LTCC localization to T-tubule membranes and plays a critical role in recruiting RyRs to the SR membrane (Figure 1). As a result, cardiac BIN1 serves as an important protein bridge for the formation and maintenance of LTCC-RyR couplons, which are essential for normal EC coupling (Zhou and Hong, 2017). Reduced myocardial BIN1 in heart failure is also detectable at the blood level, and plasma BIN1 predicts heart failure and future arrhythmias in patients with cardiomyopathy. BIN1 recruits actin to fold the T-tubule membrane, creating a “fuzzy space” that protectively restricts ion flux. When the amount of BIN1 decreases in acquired cardiomyopathy, the T-tubule morphology is altered, which contributes to dyad dysfunction and impaired EC coupling (Hong et al., 2014; Fu and Hong, 2016). Thus, a significant reduction in coupled RyR2 clusters is a result of T-tubule remodeling in a failing myocardium; where the displacement of T-tubules increases the distance to RyR and its associated protein junctophilin-2 (JPH2) and reduces the sensitivity of EC coupling and desynchrony of Ca2+ transients, typically resulting in cardiac impairment (Munro et al., 2018). Dries et al. (2018) observed a significant reduction in the density of T-tubules in patients with heart failure, with irregular distribution, and with increased T-tubule diameter and found that remodeling was associated with hyperactive spontaneous Ca2+ transient through delayed opening of non-coupled RyRs. Furthermore, JPH2 clusters attach to the SR and sarcolemma to form T-system couplons, which is linked to spatiotemporal heterogeneity of cytosolic calcium transients. A remodeling of T-tubules and a loss of couplons in the VMs of patients with heart failure leads to deleterious alterations in contractile function. Lyu et al. (2021) indicated that targeting the sarcolemmal associated JPH2 might ameliorate age-associated deficiencies in heart function. Moreover, variants of JPH2 in the joint region (mutPG1JPH2) cause T-tubule remodeling and dyad loss, resulting in asynchronous Ca2+ release (Gross et al., 2021). Thus, T-system couplons are involved in maintaining heart function by regulating the homeostasis of calcium handling. The variants of the molecules formatted T-system couplons would impair the T-tubule structure. Structural disorders of subdomains of T-system couplons provoke hyperactive Ca2+ flux, which disrupts the coordination between electrophysiological and contractile movement.

Dysfunction in T-tubules can also impair surrounding ion channels. Changes in the T-system can serve as efficient biomarkers for evaluating heart function and related prognoses. Abu-Khousa et al. (2020) observed a common feature of T-tubule remodeling in the failing myocardium. They found that the degree of T-tubule remodeling is associated with a negative cardiac force-frequency relationship and decreases contraction by reducing expression of NCX1 and restricting myocardial relaxation. Moreover, together with NCX, the T-system may be important for myocardial relaxation (Figure 1). Seidel et al. (2017) found sheet-like T-system remodeling with decreased circularity, increased volume/length ratio, and reduced t-density in patients with heart failure, which led to increased distance between RyR and sarcolemma, which also impairs the function of RyR2. Moreover, they found that the distance between RyR and sarcolemma at the time of implantation of a left ventricular assist device (LVAD) was associated with the post-LVAD left ventricular ejection fraction and contractile reverse during unloading. Frank et al. (Sachse et al., 2012) also revealed that remodeling of T-tubules could be a marker of dyssynchronous heart failure. Thyroid and glucocorticoid hormones are critical for T-system development of human induced pluripotent stem cell-derived cardiomyocytes (hiPSC-CMs) when cultured on physiological conditions (Parikh et al., 2017). Moreover, dexamethasone increases the density of T-tubules of VMs from failing human hearts, leading to improved LTCC-RyR coupling and synchrony of intracellular Ca2+ release (Seidel et al., 2019). Thus, disorders of the T-system are clinically significant, and restoring T-tubules improves the function of failing hearts.

### Molecular mechanisms of RyR2 and SERCA in Ca2+ regulation

Normal EC coupling relies on close cooperation between the SR network and T-tubule membranes. Most RyR2 clusters are located on (or close to) T-tubules, which suggests that they contribute to the generation of Ca2+ wave development during sarcomere shortening. When RyRs disordered at diastolic phase in pathological conditions, Ca2+ can leak out from the SR network and produce a Ca2+ spark. A major adverse effect of Ca2+ leak is that it reduces the amount of calcium in the SR and attenuates the amplitude of Ca2+ transient. The Ca2+ released from the SR should be taken up by SR calcium ATPase (SERCA) in every cardiac cycle to sustain a steady state with a stable Ca2+ level (Figure 1). Thus, abnormal sustained calcium leak through RyR2 or impaired reuptake by SERCA can excessively elevate cytosolic and mitochondrial Ca2+, inducing increased ROS accumulation, mitochondrial dysfunction, and even cell death.

**FIGURE 2:**
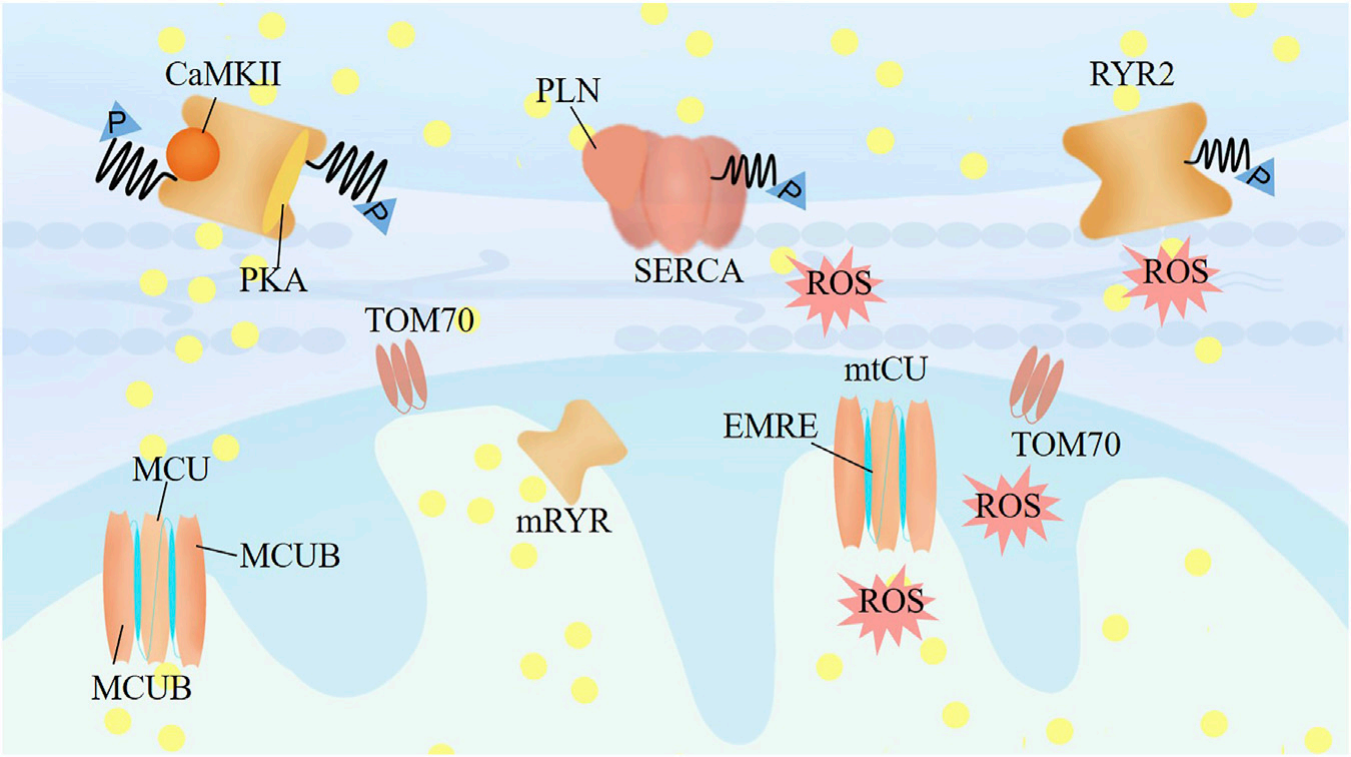
The mechanisms crosstalk between calcium and mitochondrial homeostasis. RyR2 activity is regulated by the phosphorylation level of RyR2 by the protein kinase A (PKA), Ca2+/calmodulin-dependent protein kinase II (CaMKII), and protein phosphatase type-1 and type-2A, associating with the Ca2+ level of SR. TOM70 regulates the constitutive of Ca2+ shuttling from ER to mitochondria. Mitochondrial RyR in the IMM could be activated as soon as Ca2+ released from the ER, and further increasing of mitochondrial Ca2+ would lead inactivation of mRyR1 to prevent mitochondrial Ca2+ overloading and permeability transition pore opening. Mitochondrial uptake calcium mainly through mtCU, which is a dedicated regulator for mitochondrial responding different stress to match cardiac workload with ROS and ATP production.

RyR2 activity is strongly associated with the Ca2+ level in the SR, which is also regulated by the phosphorylation of RyR2 by protein kinase A (PKA), Ca2+/calmodulin-dependent protein kinase II (CaMKII), and protein phosphatase type 1 and type 2A (Figure 2). Calcium release through RyR determines the opening time of the mitochondrial permeability transition pore (mPTP). The RyR2 channel is also regulated by S-nitrosylation and ROS production, which are mostly generated by mitochondria (Hatano et al., 2015). Yan et al. (2008) found that modification of RyR2s by ROS enhances the activity of RyR2 and increases the frequency of spontaneous Ca2+ waves. In turn, the mismatch between energy demand and supply that facilitates their transition to failing cells, and the altered calcium transfer from the SR to mitochondria, has been causally linked to the pathophysiology of aging and heart failure. Impaired mitochondria induce accumulation of glycation end products, and intracellular glycation damages the function of RyR2, leading to continuous Ca2+ leak, which in turn damages the mitochondria (Ruiz-Meana et al., 2019).

The phosphorylation of RyR2 is the dominant mechanism regulating its function, and several phosphorylation sites have been confirmed to be involved. RyR2 phosphorylation sites at S2808 and S2814, which are phosphorylated by PKA and CaMKII, respectively (Di Carlo et al., 2014), mediate diastolic SR Ca2+ leak through RyR2. Campbell et al. recently demonstrated that striated muscle preferentially expressed protein kinase (SPEG) can phosphorylate RyR2 at S2367, in contrast with previous RyR2 phosphorylation sites at S2808 and S2814. SPEG-mediated RyR2-S2367 phosphorylation can suppress diastolic SR Ca2+ leak in AF patients (Campbell et al., 2020). Another RyR2 phosphorylation site, Ser-2030, induced by PKA, is guided by integrin β1D, and integrin β1D deficiency is a novel mechanism underlying the increased risk for ventricular arrhythmias in patients with arrhythmogenic right ventricular cardiomyopathy (Wang et al., 2020).

Activation of CaMKII-mediated phosphorylation of RYR2 at S2814 can activate the latent arrhythmic potential of catecholaminergic polymorphic ventricular tachycardia (CPVT) caused by RYR2 mutations (Park et al., 2019). CaMKII is a multifunctional serine/threonine protein kinase with four major isoforms, of which CaMKIIδ is the most prevalent and relevant cardiac isoform. All CaMKII isoforms are assembled from subunits that contain three key domains: an association domain, a regulatory domain controlling enzyme activation and autoinhibition, and a catalytic domain as the kinase function of CaMKII. At rest, CaMKII is inhibited by its autoregulatory domain, which serves as a pseudo-substrate, to block the catalytic domain. Upon binding Ca2+/calmodulin (CaM), CaMKII is activated by CaM binding to its CaMKII binding site leading to T206/T207 autophosphorylation. The CaM affinity for CaMKII is increased in the sustained presence of Ca2+/CaM (Andy Hudmon and Schulman, 2002; Mustroph et al., 2017). CaMKII can also be activated by oxidation at M281/282 (Erickson et al., 2008; Yoo et al., 2018), a downstream signal of ROS modification. In contrast, oxidation of M308 by molecules interacting with microtubule associated monooxygenase, calponin, and LIM domain containing 1 (MICAL1), a methionine monooxygenase, can reduce Ca2+/CaM binding and prevent pathological CaMKII activation (Konstantinidis et al., 2020).

CaMKII can also phosphorylate phospholamban (PLN) to further activate SERCA, which is important in the uptake of Ca2+ from the cytoplasm to the SR (Maier and Bers, 2007). Decreased SERCA activity is associated with impaired diastolic function. Oxidative modification at cysteine 674 (C674) of SERCA at the post-translational level can decrease SERCA activity (Lancel et al., 2009; Qin et al., 2013). The presence of an oxidation-resistant mutant of SERCA (C674S) can protect cardiomyocytes from mitochondrial calcium overload and cardiomyocyte death by mitochondria-dependent pathways (Goodman et al., 2020). The c-jun N-terminal kinases (JNKs), which are activated in response to stress, can be activated in various cardiovascular diseases, including cardiac arrhythmia and heart failure. JNK2 can activate the transcription factor of c-jun, which can further activate transcription factor 2 (ATF2) binding to the proximal promoter region (Gao et al., 2018). Moreover, the activation of JNK2 can directly phosphorylate CaMKII (Yan et al., 2018a; Yan et al., 2018b), enhancing the release of calcium from RyR2 and increasing the risk for arrhythmia. JNK2 can directly elevate the max velocity of SERCA2 activity by phosphorylation (Yan et al., 2021), protecting the heart from arrhythmia and maintaining calcium regulation. A-kinase anchoring protein (AKAP18δ) can be an anchor of CaMKIIδ that directly regulates CaMKIIδ activity which would inhibit the faster Ca2+ reuptake by PLN-SERCA2 or Ca2+-release through RYR (Carlson et al., 2021).

The mechanism underlying Ca2+ handling is intricate. Ca2+ overload in the cytosol increases the affinity of troponin binding Ca2+, contributing to arrhythmogenicity and heart failure. Elucidating the precise mechanisms underlying calcium handling intracellularly and downstream of mitochondrial function is important for understanding the causes and progression of cardiomyopathy and developing novel and precise strategies for treating cardiomyopathy.

### Crosstalk between cardiac dyads and mitochondrial homeostasis in calcium handling

The function of cardiac dyads was tightly correlated with mitochondrial. The metabolic status and ROS regulation mediated the functional compacts of cardiac dyads. Besides, communications had been identified between cardiac dyads and mitochondrial in calcium uptakes and transits. Cardiomyocytes rely heavily on mitochondria to meet energy demands during bioenergetic EC coupling and mitochondrial buffering of Ca2+. Rog-Zielinska et al. (2019) observed T-tubules and mitochondria were directly connection, the distance between of them was around 18.05 nm. The molecular mechanisms between T-tubules and mitochondria are not clarified clearly. Under a cellular global Ca2+ release from the SR, mitochondria are frequently exposed to intracellular spatiotemporal increases in Ca2+, leading to an increase in Ca2+ in mitochondria. A significant increase in mitochondrial Ca2+ *via* the mitochondrial calcium uniporter (mtCU) reduces the mitochondrial membrane potential, potentially inducing ROS generation *via* perturbation of the pH gradient at the inner mitochondrial membrane (IMM), affecting the rate of mitochondrial energy production, mitochondrial motility, and morphology (Figure 1). (Garcia-Perez et al., 2008; Liu et al., 2014; Santulli et al., 2015; Lesnefsky et al., 2017; Wust et al., 2017) Mitochondrial ROS can promote SR calcium leak through CaMKII-dependent RyR2 modification. Thus, ROS regulation is considered as a dominant feature in mitochondrial related calcium handling. While the Ca2+ communication and transits between cardiac dyads and mitochondria *via* transmembranous channels are also critical for calcium homeostasis. Excessive SR calcium leak contributes to mitochondrial calcium overload, leading to mitochondrial dysfunction. There is a maladaptive positive feedback relationship between these closely associated organelles when cardiomyocytes are damaged.

Duchen et al. observed that focal SR calcium release results in calcium microdomains sufficient to promote local mitochondrial calcium uptake, which suggests a tight coupling of calcium signaling between SR release sites and nearby mitochondria (Michael et al., 1998). Studies have proven physical interaction between the SR and mitochondria and demonstrated that the distance between the SR and mitochondria is approximately 10–25 nm (Saverio Marchi and Pinton, 2014; Csordas et al., 2018). The rapid development of biochemical techniques enables the isolation of SR-mitochondria binding sites, also known as mitochondria-associated SR membranes (MAMs). Moreover, it also enables the identification of various proteins that reside within MAMs and illustrates the functional interaction between the SR and mitochondria. Mitofusin 2 (MFN2), a mitochondrial dynamin-related protein, is enriched at the SR-mitochondria interface. The ablation or silencing of MFN2 suppresses SR-mitochondria interactions and attenuates the efficiency of mitochondrial Ca2+ uptake (Olga Martins de Brito and Scorrano, 2008; Chen et al., 2012). Paillard et al. found CypD at the MAMs was important in adjusting mitochondrial Ca2+ by interacting with VDAC1/Grp75/IP3R1 complex (Paillard et al., 2013). The inhibition of CypD in adult cardiomyocytes decreased the Ca2+ transfer from SR to mitochondria through IP3R under normoxic conditions. Moreover, Qi et al. (2021) presented that Sphingosine-1-phosphate (S1P) mediated the intracellular Ca2+ signaling, by regulating SR-mitochondria communication *via* IP3R2 in cardiomyocyte hypertrophy. Mitochondrial Ca2+ uptake plays an essential role in the regulation of numerous cellular processes, including energy metabolism and cytosolic Ca2+ homeostasis interacting with cardiac dyads.

The translocase of the outer membrane (TOM) consists of a large proportion of the outer mitochondrial membrane (OMM) proteome. TOM70 puncta are frequently associated with MAMs, which regulate Ca2+ shuttling from the SR to mitochondria (Figure 2). Filadi et al. (2018), Beutner et al. (2005) characterized the mRyR in the IMM of rat heart as RyR1 and found that mRyR1 can be activated as soon as Ca2+ is released from the SR. A further increase in mitochondrial Ca2+ leads to inactivation of mRyR1 to prevent mitochondrial Ca2+ overload and opening of the permeability transition pore. Additionally, mitochondrial Ca2+ is a crucial modulator of mitochondrial permeability transition, and a sudden increase in Ca2+ in IMM permeability dissipates ΔΨm and releases ROS and cytochrome C (Hausenloy et al., 2004). The entry of calcium through the mtCU is the central mediator of mPTP opening, although the precise molecular makeup of the mPTP remains elusive. Mitochondria take up calcium mainly through the mtCU, a multiprotein complex (≈700 kDa) located in the IMM, which is composed of pore-forming proteins (the channel subunit MCU and the MCU dominant-negative β subunit [MCUB]), the short transmembrane regulator EMRE (an essential MCU regulator), and regulatory proteins (mitochondrial calcium uptake proteins [MICU] 1, 2, and 3 and mitochondrial calcium uniporter regulator 1 [MCUR1]; Figure 2). (Kaludercic and Scorrano, 2019; Alevriadou et al., 2021) The uptake of calcium into the mitochondria by MCU is a dedicated regulator of the mitochondrial response to stress to match cardiac workload with ATP production (Kwong et al., 2015).

Ru360, a specific inhibitor of the mtCU, can decrease mitochondrial calcium dramatically and prevent mPTP opening after cardiac ischemia (Garcia-Rivas Gde et al., 2006). Pan et al. (2013) found a significant reduction in, but not completely absence of, mitochondrial matrix calcium in mice lacking MCU, which resulted in a loss of stress-responsive signaling. A recent study have found that MCUB expression can displace MCU from the functional mtCU complex, reducing the association between MICU1/2 and mitochondrial calcium uptake, a stress-responsive mechanism to limit mitochondrial calcium overload during cardiac injury (Lambert et al., 2019). Overexpression of MCUB can inhibit mitochondrial calcium uptake in cardiomyocytes and partially protect cardiomyocytes from ischemia-reperfusion injury by reducing mPTP opening (Huo et al., 2020). However, a loss of MICU3 can reduce calcium loading during sustained treatment with isoproterenol (Puente et al., 2020). Moreover, mitochondrial calcium uptake can regulate the epigenome and influence cellular differentiation and maturation in an MICU1-dependent fashion (Shanmughapriya et al., 2018; Lombardi et al., 2019). Barth syndrome (BTHS), which is caused by a mutation in the Tafazzin (TAZ) gene, encodes an acyltransferase catalyzing the remodeling of cardiolipin in mitochondrial membranes (Clarke et al., 2013). Mice deficient in TAZ have lower expression of MCU in cardiomyocytes, which suppresses Ca2+ uptake in mitochondria, resulting in a lack of Ca2+-induced Krebs cycle activation (Bertero et al., 2021). Joiner et al. (2012) showed that CaMKII promoted mPTP opening and myocardial death by increasing the mtCU current and identified CaMKII activity as a central mechanism for mitochondrial Ca2+ entry. However, Fieni et al. (2014) found that the mtCU current is not regulated by CaMKII directlydirectly. The intricacy of the mtCU and the complexity of regulation of mtCU calcium uptake require further elucidation.

In sum, the crosstalk between mitochondria and SR Ca2+ handling in cardiomyocytes is increasingly being recognized. The mechanism underlying mitochondrial Ca2+ handling and suitable amounts of mitochondrial Ca2+ on a beat-by-beat basis in normal and damaged cardiomyocytes are unclear. It is thought that the structure of dyads plays an important role in cardiomyocytes, especially in the efficiency of calcium release during EC coupling.

### Treatment basis Ca2+ handling and mitochondrial homeostasis

Therapeutic strategies have been designed to increase the contractile force of the heart to directly improve the survival of patients with severe heart failure. Although a series of genetic analyses and mechanistic studies have revealed the crosstalk between calcium handling and mitochondrial homeostasis as the central mediator of ventricular arrhythmia and heart failure, the FDA has yet to approve reagents targeting calcium handling and mitochondrial homeostasis for use by cardiovascular patients. Coldren et al. (2020) used high-throughput virtual screening combined with concurrent stopped-flow kinetic experimental verification to identify a number of sensitizers that slowed the calcium off-rate; they expected these Ca2+ sensitizers would have therapeutic potential for heart disease in the near future. Fiedler et al. (2019) invented a small-molecule inhibitor, DMX-5804, to limit activation of mitogen-activated protein kinase-4 (MAP4K4), which can reverse mitochondrial function and calcium cycling to improve the survival of hiPSC-CMs. Another small molecule of ARM036, which is able to stabilize the closed conformation of RyR2, helped mitigate the increased sensitivity of RyR2 under low resting Ca2+ and maintain normal LV dimensions and ejection fractions in a young dog model of Duchenne muscular dystrophy (Olivier Cazorla et al., 2021). Joiner et al. (2012) found exogenous expression of a membrane-targeted CaMKII inhibitor in mice was able to mitigate cardiac ischemia-reperfusion injury by inhibiting IMCU. A recent study found cardiac-specific overexpression of MCU maintained intracellular Ca2+ homeostasis and contractility, which is a compensatory mechanism that counteracts stress-induced pathological cardiac remodeling by preserving Ca2+ homeostasis and cardiomyocyte viability (Wang et al., 2022). Metformin can stabilize the structure of MAMs, reduce the expression of MICU1, and lower the amount of mitochondrial Ca2+, which enhances the function of respiration chain complex I in dilated cardiomyopathy (Angebault et al., 2020). Recently, Mustroph et al. (2022) demonstrated the SLGT2i, Empagliflozin, could inhibited cardiac late sodium current by CAMKII. Besides, Zhang et al. (2022) demonstrated the Hesperadin could serve as the CaMKII specific inbitor to ameliorate cardiac ischemia/reperfusion injury.

Pu and his colleagues have observed calcium mishandling and mitochondrial remodeling in BTHS (Liu et al., 2021) and a CPVT model (Bezzerides et al., 2019). They showed that a selective CaMKII autocamtide-2-related inhibitory peptide transducing with adeno-associated virus (AAV) can correct BTHH and CPVT Ca2+ transient amplitude and reduce diastolic Ca2+ concentrations to a base level, resulting in the suppression of arrhythmias in murine models. Schweitzer et al. (2017) demonstrated that enhanced mitochondrial Ca2+ uptake after treatment with Efsevin, a synthetic agonist of voltage-dependent anion channel two in the OMM, suppressed arrhythmia in a murine model of CPVT. Besides the AAV vector-based gene therapy, several non-viral vectors or strategies had been applied for inherit cardiovascular diseases. Nanoparticles had been introduced to gene therapy for several years, as it presented advantages in effective compositions, especially for lipid nanoparticles (LNP) (Godbout and Tremblay, 2022). Experiments proved LNPs could be delivery RNAs to brain, lungs, heart, liver and bones. Moreover, the self-assembled nanostructured particles from natural building blocks, including polyphenol materials, could also be used for gene therapy directly or *via* cell-based modification to delivery oligonucleotide (Ju et al., 2022).

Evidence of these molecules and their therapeutic impacts on cardiac diseases reveals two novel methods of delivering these molecules to cardiomyocytes: viral and non-viral strategies. Viral strategies involve the use of several vectors, including adenovirus, AAV, and lentivirus. Non-viral strategies involve using liposomes or nanoparticles to deliver oligonucleotides to heart tissue. At present, more than 30 clinical trials of gene therapy are under way involving diseases of the brain, spinal cord, eye, liver, and muscle. Taking advantage of various delivery vectors targeting calcium and mitochondrial homeostasis could terminate or even reverse pathological remodeling in cardiomyopathy, which would have the effect of acting as an isogeneic implantable cardioverter defibrillator (ICD).

## Future aspects

Over the last decades, a large amount researches expanded our knowledge of the Ca2+ handling among the interplays of T-tubule, ER, and mitochondrial in cardiomyocytes. Yet, there are also some unsolved queries and conflicting experimental results. It is urgent to explore the factors involved in coordinating formation of the T-tubular network, along with the potential molecular mechanisms in maintaining homeostasis of intricate architecture between T-tubule and the ER. Moreover, it is also critical to illustrate the transcriptional regulation mechanisms of various proteins reside within the MAMs and the precise mechanisms of calcium handling between ER and mitochondria. Besides, the balance between sufficient ATP consumption and acceptable ROS generation is still required further study. The further understanding of the molecular mechanisms involved in the crosstalk between Ca2+ handling and mitochondria in cardiomyocytes may provide newly concepts on the treatment of cardiovascular disease. And isogeneic therapeutic strategy would be another alternation to handle deadly arrhythmia and improve their prognosis.
